# Case Report: Transient perivascular inflammation of the carotid artery syndrome: a rare etiology of self-limiting carotidynia

**DOI:** 10.3389/fimmu.2026.1698493

**Published:** 2026-03-13

**Authors:** Yan Meng, Jie Zhang, Li-yang Bai, Chun-yuan Huo, Lijuan Wang

**Affiliations:** 1 Neuroscience Center, Department of Neurology, First Hospital of Jilin University, Jilin University, Changchun, China; 2 Department of Electrodiagnosis, Changchun Central Hospital, Changchun, China

**Keywords:** carotid artery diseases, immunity, self-limiting disease, transient perivascular inflammation, ultrasound

## Abstract

**Objectives:**

Transient perivascular inflammation of the carotid artery (TIPIC) syndrome is a recently recognized clinicoradiological entity that has not yet been formally incorporated into established disease classifications. It is frequently overlooked in clinical practice because of its unclear etiology and nonspecific symptoms. This study aimed to provide a comprehensive summary of the existing literature to improve clinical awareness and diagnostic accuracy.

**Methods:**

A retrospective analysis of literature on TIPIC syndrome published before May 2024 was performed, while we reported a case of TIPIC syndrome. The results of both were collated and discussed.

**Results:**

A total of 58 reports including 137 patients with TIPIC were identified. The median age of the patients was 48 years, with a female predominance. All patients presented with neck pain or other concomitant symptoms, mostly with unilateral involvement. All patients had a characteristic imaging presentation with eccentric inflammatory infiltrates in the vessel wall and surrounding tissues. About 1/3 of the patients had luminal stenosis but no blood flow disturbances. The median time to resolution of neck pain symptoms in patients was 2 weeks. Both clinical symptoms and imaging abnormalities may resolve spontaneously or following pharmacological treatment.

**Conclusions:**

TIPIC syndrome appears to be a self-limiting condition that may involve transient immune-mediated mechanisms. Clinical awareness of this disease is needed to prevent missed diagnosis or misdiagnosis. The role of imaging in differential diagnosis and follow-up should be appreciated. There are still many unknowns in TIPIC syndrome and a large number of clinical studies are needed to fill these gaps.

## Introduction

Carotidynia was first described and named by Fay in 1927 as a syndrome characterized by pain at the carotid bifurcation (1). In 1988, carotidynia was classified by the International Headache Society (IHS) in the first International Classification of Headache Disorders (2). However, some scholars considered carotid pain to be a painful symptom rather than a disease (3) and the symptoms were not entirely consistent with the diagnostic criteria indicated by the IHS in 1988 (4). It was subsequently removed from the IHS classification as an entity in 2004 (5). Due to the scarcity of cases and the tendency for this symptom of carotid pain to go unnoticed, there has been a lack of systematic research on carotidynia, which has remained a vague concept. It was not until 2017 that Lecler et al. replaced this vague entity with a new term: transient perivascular inflammation of the carotid artery (TIPIC) syndrome, through a multicenter study of 47 patients, all of whom had acute neck pain and similar imaging presentations of vascular and peripheral abnormalities (1). This entity has since received increasingly widespread attention, with several scholars clearly capturing the characteristic manifestations which present as eccentric perivascular inflammatory infiltration, by means of magnetic resonance imaging (MRI), computed tomography (CT), and ultrasound. However, awareness of TIPIC among clinicians remains limited.

TIPIC rarely causes hemodynamic stenosis and has many similarities with other vascular diseases such as atherosclerotic plaques, arteritis, and arterial dissection. So it is easily missed or misdiagnosed. Currently, there is relatively little research on TIPIC, and most of them are case reports or small sample size retrospective studies. Its natural history, etiology, best imaging tool, morphological characteristics, and appropriate treatment methods are still unclear (6). Hence, we performed a review of literature on existing studies on TIPIC and present a case of a young man with TIPIC, initially diagnosed with Takayasu arteritis, and later confirmed to be TIPIC through follow-up in order to enhance clinical doctors’ understanding of this disease. Written informed consent for patient information and images to be published was obtained.

## Case report

**Figure 1:**
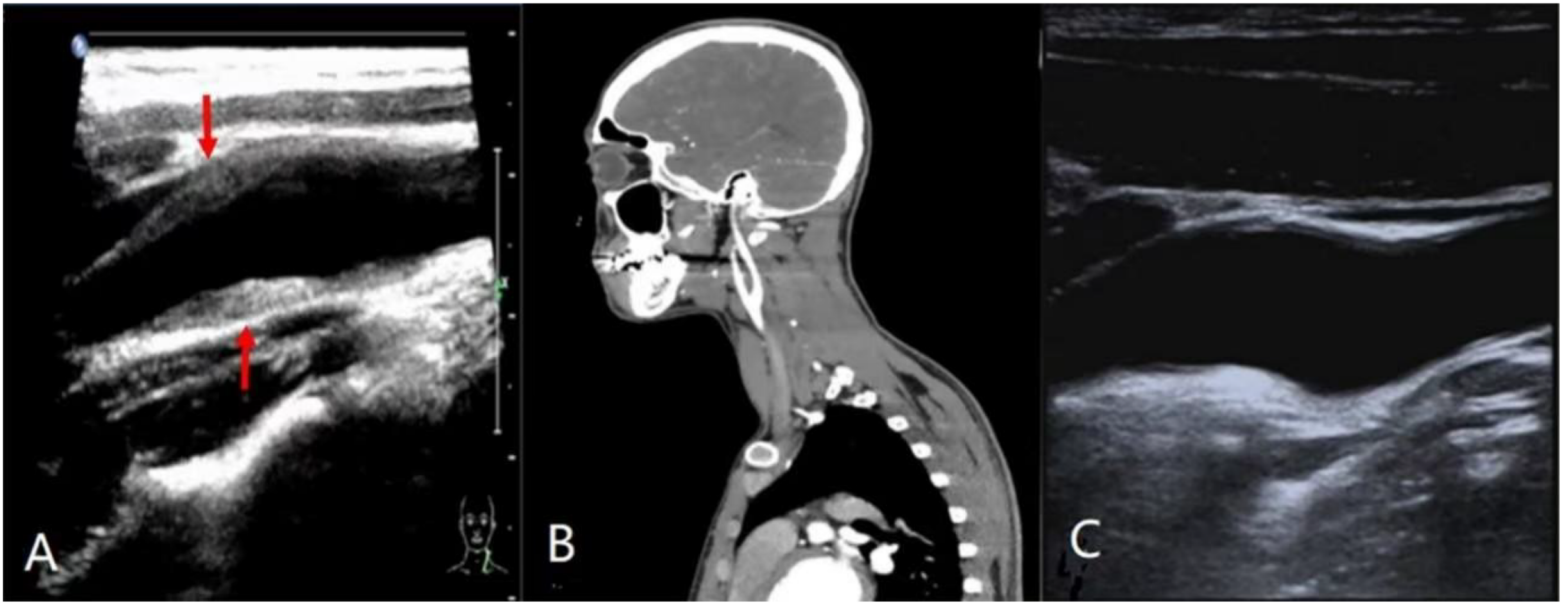
Imaging presentation of a patient with TIPIC syndrome: **(A)** Diagnostic ultrasound shows a bifurcation of the carotid artery with a deviated perivascular infiltrate (arrow), the presence of a mild luminal stenosis, no hemodynamic changes. **(B)** Patient underwent Computed Tomographic Angiography a few days after treatment, suggesting complete normalcy. **(C)** Ultrasound follow-up after one month shows disappearance of eccentric infiltrates in the carotid bifurcation, with lumen patency restored.

A 22-year-old Chinese male presented with a week-old episode of left-sided neck pain with bilateral mandibular lymph node swelling of no apparent cause, which progressively worsened and radiated to the head and pharynx four days later, and was treated with antibiotics to no avail. The patient was previously fit and had only a history of appendicitis surgery. He was initially admitted to the hospital and various tests were carried out, the body temperature was normal with no fever. On examination the patient had a normal pulse and no difference in blood pressure in both upper limbs. Palpation of the patient’s neck revealed significant tenderness and pressure, no swelling, and normal carotid artery pulsation without murmurs. Laboratory investigations revealed significantly elevated erythrocyte sedimentation rate (ESR), C-reactive protein (CRP), and serum immunoglobulin M (IgM) levels. The patient then underwent vascular ultrasound. The large abdominal vessels and bilateral upper and lower limb arteries were normal, but the carotid ultrasound revealed a deviated perivascular infiltrate at the left carotid bifurcation, with a maximum thickness of 3.2 mm. The lumen was mildly stenosed, but there was no obstruction to blood flow. The patient was initially diagnosed with Takayasu arteritis, and systemic corticosteroid therapy was initiated. One week later, the patient’s pain symptoms alleviated and computed tomography angiography (CTA) examination showed no significant abnormalities at the carotid artery. One month later, the patient’s symptoms disappeared, and the deviated perivascular infiltrate at the left carotid bifurcation also disappeared on carotid ultrasound. Based on the self-limiting clinical course and imaging resolution, the diagnosis was revised from aortitis to TIPIC syndrome. After 6 months, the patient showed no symptoms and no abnormalities were found on carotid ultrasound (**Figure 1**).

### Clinical timeline

The chronological sequence of clinical events is summarized in **Table 1**.

**Table 1:** Clinical timeline of the reported case.

Time point	Clinical events
1 week before admission	Onset of left-sided neck pain with bilateral mandibular lymph node swelling
Day 4 after onset	Pain worsened and radiated to the head and pharynx
Before admission	Empirical antibiotic therapy administered without clinical improvement
Admission (Day 0)	Physical examination revealed focal tenderness over the left carotid region; normal vital signs and no blood pressure asymmetry
Day 0	Laboratory tests showed elevated ESR, CRP, and serum IgM levels
Day 1	Carotid ultrasound demonstrated eccentric perivascular infiltrate at the left carotid bifurcation (maximum thickness 3.2 mm) with mild luminal stenosis but preserved flow
Day 1	Initial diagnosis of Takayasu arteritis; corticosteroid therapy initiated
1 week after treatment	Marked pain relief; CTA showed no significant luminal abnormalities

### Patient perspective

The patient reported significant anxiety at the time of initial diagnosis due to concern about potential systemic vasculitis. Following symptom resolution and revision of the diagnosis to TIPIC syndrome, he expressed reassurance and confidence in the conservative management approach. He consented to continued follow-up.

### Literature review

A systematic literature search was performed in PubMed/MEDLINE using the search terms “TIPIC syndrome”, “Carotidynia”, and “Idiopathic carotiditis”, including articles published in English up to May 2024. A total of 190 records were identified. Duplicate screening based on PubMed identification numbers (PMIDs) revealed no duplicate entries. All 190 records underwent title and abstract screening. The review question was structured according to the PICO framework: Population (patients diagnosed with TIPIC syndrome or idiopathic carotidynia), Exposure (imaging-confirmed perivascular carotid inflammation), Comparator (not applicable), and Outcomes (clinical presentation, imaging findings, treatment, and recurrence).

Studies were excluded if they were review articles, classification or guideline papers, large-vessel vasculitis (e.g., Takayasu arteritis or giant cell arteritis), carotid dissection, tumor-related carotid pathology, secondary inflammatory conditions, basic research, or lacked sufficient clinical or imaging data. Articles for which the full text was unavailable were also excluded.

After screening, 132 records were excluded. The remaining 58 articles met the predefined inclusion criteria, namely case reports or case series describing patients with TIPIC syndrome or idiopathic carotidynia supported by characteristic imaging findings. All included studies were derived exclusively from the original PubMed search results. Ultimately, 58 articles comprising 137 patients were included in the qualitative synthesis (**Table 2**).

**Table 2:** Literature review.

Author	Patients	Symptom	Modality	Treatment
Polich A et al (6)	1, 50F	Neck pain and tenderness	Ultrasound and MRI	Untreated
Sanghvi DA et al (27)	1, 46F	Neck pain, tenderness and swelling	MRI	NSAIDs
Buetow MP, Delano MC (28)	1, 49M	Neck pain and swelling	MRI	Not reported
Hemmen TM, Bettle N, Borelli AJ Jr (29)	1, 42M	Neck pain and tenderness	MRI	NSAIDs
Stanbro M Gray BH, Kellicut DC (26)	3, 65F 42F 38M	Neck pain and tenderness	Ultrasound and MRI	NSAIDs and Sedative
Hayashi S et al (30)	1, 64M	Neck pain and tenderness	Ultrasound, CT and MRI	NSAIDs
Hersh SP Gerard P, Hersh J (31)	1, 74F	Neck pain and tenderness, radiated to the ipsilateral face	CT	Not reported
Parra A Okada T, Lin PH (11)	2, 55M 56F	Neck pain and tenderness	Ultrasound and MRI	NSAIDs
Abrahamy M et al (32)	6, 47M 37M 41F 71F 52F 57F	Neck pain and tenderness	Ultrasound and MRI	NSAIDs
Burton BS et al (33)	5, 48M 26M 31F 51F 51F	Neck pain and tenderness	MRI	NSAIDs
Comacchio F et al (34)	1, 70M	Neck pain, tenderness and swelling	Ultrasound and MRI	NSAIDs
Sena LA et al (35)	1, 73F	Neck pain and tenderness, radiated to the preauricular face	MRI	Steroids
Coulier, B Van den Broeck S, Colin GC (36)	1, 55F	Neck pain	Ultrasound and MRI	NSAIDs
Cassone G et al (37)	1, 31F	Neck pain and tenderness, radiated to the ipsilateral ear, eye, and occipital	MRI	NSAIDs
Inatomi Y et al (38)	1, 44F	Neck pain, tenderness and swelling	Ultrasound and MRI	Steroids
Kosaka N et al (39)	4, 53M 46F 47F 59F	Neck pain	Ultrasound and MRI	Not reported
Del Conde I, Baumann F (40)	1, 47M	Neck pain and tenderness	Ultrasound and MRI	Steroids
Santarosa C et al (41)	1, 34M	Neck pain	Ultrasound and MRI	NSAIDs
Behar T et al (42)	1, 52M	Neck pain	Ultrasound and MRI	NSAIDs
Andersen TT et al (43)	1, 47M	Neck pain	Ultrasound	Antibiotic
da Rocha AJ et al (44)	1, 52M	Neck pain and tenderness	Ultrasound and MRI	Steroids
Schaumberg J, Eckert B, Michels P (45)	1, 49F	Neck pain, radiated to the chin and ear	Ultrasound and MRI	Not reported
Tardy J et al (46)	1, 30F	Neck pain and tenderness, radiated to the ipsilateral ear	Ultrasound and MRI	NSAIDs
Park JK et al (10)	1, 49F	Neck pain, radiated to the ipsilateral ear and frontotemporal area	MRI	Analgesics
Lecler A et al (1)	18, M 27, F	Neck pain, tenderness and swelling	Ultrasound, CT and MRI	NSAIDs, Steroids and untreated
Ulus S et al (47)	15, 27M 32F 38F 39M 39M 39M 39M 39F 41F 45F 46F 46M 55F 61F 62M	Neck pain and tenderness	Ultrasound and MRI	NSAIDs, Steroids, Analgesics, Antibiotic and untreated
Kuhn J et al (48)	1, 49F	Neck pain, tenderness and swelling, radiated to the chin and ear	Ultrasound and MRI	NSAIDs
Amaravadi RR et al (7)	1, 68F	Neck pain	18F-FDG PET, MRI and CTA	Untreated
Young JY, Hijaz TA, Karagianis AG (15)	1, 70F	Neck pain	CT	Untreated
Berzaczy D et al (49)	1, 52M	Neck pain and swelling	Ultrasound	NSAIDs
Lee TC et al (50)	1, 51F	Neck pain and swelling	MRI, MRA and CTA	Untreated
Scoppettuolo P et al (51)	1, 35F	Neck pain and tenderness	Ultrasound and CTA	NSAIDs
Chen XW et al (16)	1, 71F	Neck pain and tenderness, vocal hoarseness, choking and coughing	MRI	Steroids
Azar L, Fischer HD et al (12)	1, 59F	Neck pain and tenderness	CT	Steroids
Woo JK et al (52)	1, 46M	Neck pain	Ultrasound, CTA and MRI	Steroids
Richier Q, Travers JY, Raffray L et al (53)	1, 34M	Neck pain	Ultrasound and CT	Not reported
Rafailidis V et al (17)	3, 40M 52M 68F	Neck pain, radiated to the ipsilateral ear	Ultrasound and CTA	NSAIDs
Modi T et al (54)	1, 35F	Neck pain and swelling	Ultrasound and MRI	Steroids
Syms MJ, Burton BS, Burgess LP (55)	1, 48M	Neck pain and swelling	MRI	Untreated
Venetis E, Konopnicki D, Jissendi Tchofo P (56)	1, 38M	Neck pain, radiated to the mandibular angle	Ultrasound, CT and PET	Untreated
El Nawar R et al (57)	1, 52F	Neck pain	Ultrasound and MRI	Untreated
Maggialetti N et al (58)	1, 49M	Neck pain and swelling, radiated to the ipsilateral ear	Ultrasound and MRI	NSAIDs
Mathangasinghe Y, Karunarathne RU, Liyanage UA (59)	1, 43F	Neck pain and tenderness	Ultrasound and MRI	NSAIDs
Takamura A, Hori A (60)	1, 44M	Neck pain and tenderness, radiated to the ipsilateral ear and lower jaw	Ultrasound	Untreated
Lecler A, Obadia M, Sadik JC (61)	1, 31F	Neck pain	Ultrasound	Steroids
Czihal M et al (18)	5, 36F 44F 55F 73F 42M	Neck pain and swelling	Ultrasound	Steroids
Mumoli N et al (25)	1, 36M	Neck pain, tenderness and swelling	Ultrasound and MRI	Antibiotic
Skalla E et al (62)	1, 35M	Neck pain	Ultrasound	Untreated
Badou E et al (63)	1, 48M	Neck pain	Ultrasound and MRI	Untreated
Greutert S, Schlomer T, Righini M (64) Peycheva M et al (65) Ulus S, Denizoğlu N, Akarçay M (14) Holay Q, Hak JF, Varoquaux A (66) Abreu JA et al (67) Arnould B et al (13) Sandu GC et al (68) Daoussis D et al (69)	1, 37F 1, 52M 1, 39M 1,46F 1, 57F 1, 73F 1, 49F 1, 62F	Neck pain and tenderness Neck pain and tenderness Neck pain Neck pain and tenderness Neck pain, tenderness and swelling Neck pain, tenderness and swelling Neck pain Neck pain	MR Ultrasound and CTA Ultrasound CT and MRI Ultrasound and CT Ultrasound and CT Ultrasound, CT, CTA and MRI PET/CT	NSAIDs NSAIDs NSAIDs Untreated NSAIDs Antibiotic NSAIDs and Steroids NSAIDs

F, Female; M, Male; MRI, Magnetic Resonance Imaging; CT, Computed Tomography; CTA, Computed Tomographic Angiography; NSAIDs, Non-steroidal Anti-Inflammatory Drugs, FDG, Flurodeoxyglucose; PET, Positron Emission Tomography.

This literature review was conducted and reported in accordance with the Preferred Reporting Items for Systematic Reviews and Meta-Analyses (PRISMA) 2020 statement.

**Figure 2:**
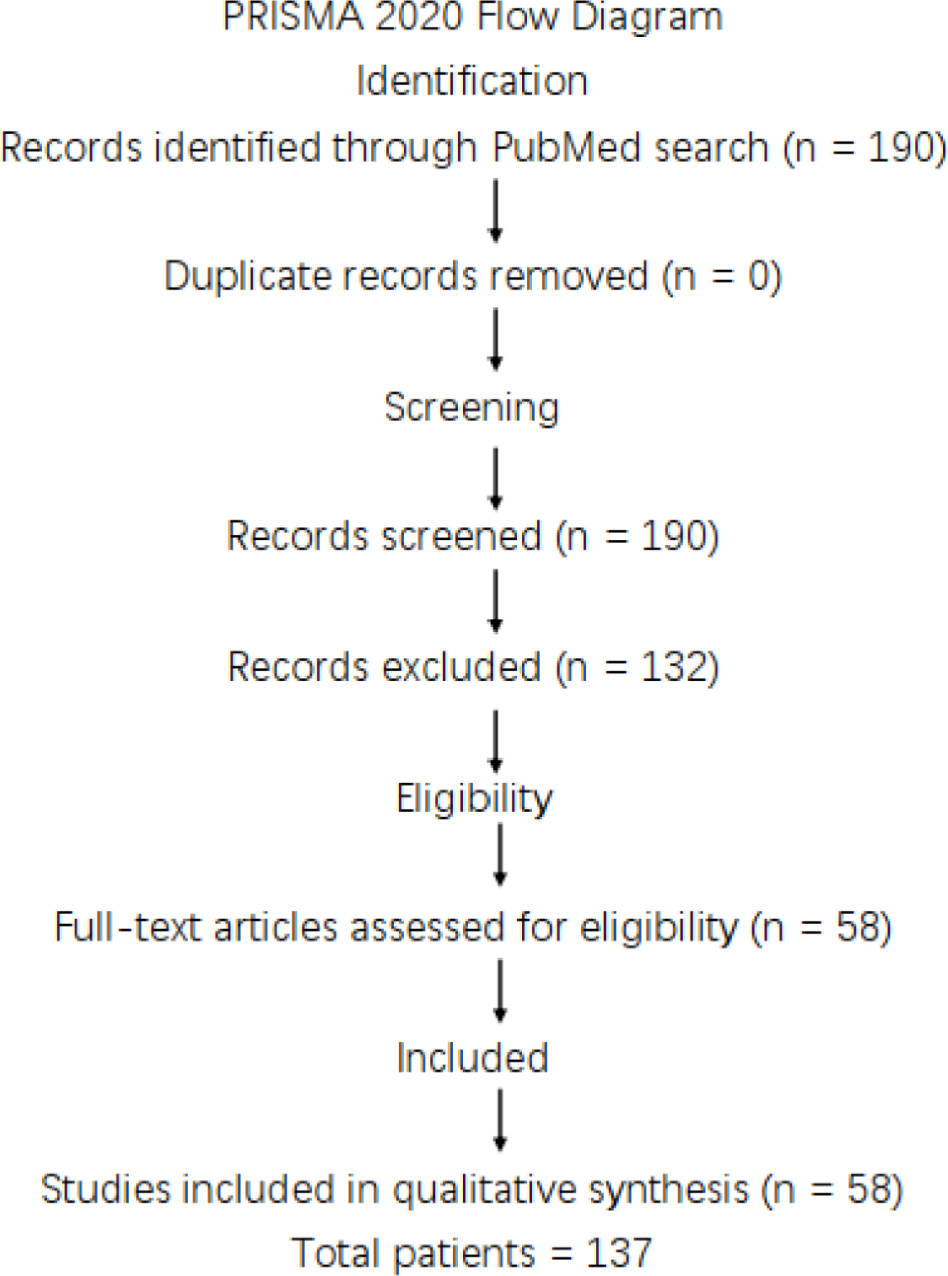
PRISMA 2020 flow diagram of study selection. Flow diagram illustrating the identification, screening, eligibility assessment, and inclusion of studies in the systematic literature review. A total of 190 records were identified through PubMed search. After screening and application of predefined eligibility criteria, 58 case reports comprising 137 patients were included in the qualitative synthesis.

**Figure 2** PRISMA 2020 flow diagram of study selection.

## Results

Some reports lacked complete clinical data. To minimize bias, data extraction was performed independently by two reviewers.

### Characteristics of patients

A total of 137 patients were included in this review, including one of our patients. The male/female ratio of these patients was about 1:1.40 (57/80) and the median age of the patients was 48 years, with a minimum of 22 years and a maximum of 74 years. All patients presented with neck pain. A total of 133 patients specified whether there was pressure and swelling in the neck, of which 94 patients (70.7%) had pressure and 21 patients (15.8%) had swelling. A total of 128 patients specified whether the pain radiated, of which 13 patients (10.1%) radiated to other parts of the neck or areas of the head such as the face, behind the ears and occipital. A total of 128 patients specified whether there was lymph node enlargement, and 12 patients (9.4%) had lymph node enlargement. A total of 131 patients had a clear history of previous medical or vascular risk factors, and 46 patients (35.1%) had vascular risk factors, including 25 (19.1%) had smoking, 18 (13.7%) had dyslipidemia, 16 (10.4%) had hypertension and 3 (2.3%) had diabetes mellitus. Three patients (2.3%) presenting with flu-like symptoms of upper respiratory tract infection and 3 (2.3%) had fever. Four patients (3.1%) had cancers of the lung and breast. Twelve patients (9.2%) had a history of autoimmune disease, including 2 cases of ankylosing spondylitis, 1 case of Hashimoto’s thyroiditis, 1 case of Graves’ disease, 1 case of systemic lupus erythematosus, 1 case of Sjogren’s syndrome, 3 cases of rheumatoid arthritis, 1 case of antiphospholipid syndrome and 1 case of multiple sclerosis. Four patients (3.1%) had a history of hematological disease: 2 myelodysplastic syndrome and 2 leukemia. Eight patients (6.1%) had a viral infection, 1 patient (0.8%) had a history of neck trauma, 1 patient (0.8%) had a tuberculosis infection and twelve patients (9.2%) presented with associated neurological symptoms. A total of 110 patients had definite biochemical findings, of which 83 patients (75.5%) had normal tests, 27 patients (24.5%) had elevated erythrocyte sedimentation rate and C-reactive protein, 3 patients (2.7%) had elevated immunoglobulin-M. The site of involvement was clearly identified in 135 patients, with 72 patients (53.3%) having left-sided involvement, 57 patients (42.2%) having right-sided involvement and 8 patients (5.9%) having bilateral involvement.

### Diagnostic imaging data

All 137 patients were observed with a characteristic presentation on ultrasound, MR and CT images by one or more means. In total, 144 carotid arteries were involved, with the majority of lesions located posteriorly (48.6%) and laterally (47.9%). Of the 136 vessels with a specific lesion location, a total of 99 vessels (72.8%) occurred in the carotid bifurcation, rarely located in the distal common carotid artery, proximal external carotid artery and proximal external carotid artery. Of the 129 vessels for which hemodynamics were specified, 34 vessels (26.4%) showed luminal stenosis mildly and 1 vessel (0.8%) showed luminal stenosis moderately, the remaining vessels showed no significant luminal obstruction. Plaques were present in 30 of the 126 vessels (23.8%) for which the presence or absence of plaque was clarified.

### Treatment and prognosis

There were 127 patients had a clear treatment plan, with 79 patients (62.2%) receiving anti-inflammatory drugs and 23 patients (18.1%) receiving steroids, 6 (4.7%) with antibiotics, 2 (1.6%) with sedative-hypnotic drugs, 2 (1.6%) with analgesics and 24 (18.9%) were cured without any treatment. The median time to resolution of symptoms was 2 weeks. In total, 130 patients were clarified as to whether they had a relapse, of which 14 patients (10.8%) had a recurrence at the original side of the lesion or on the opposite side of the lesion after they had recovered.

## Discussion

Our study reported an initial misdiagnosed case of TIPIC and performed a literature review about the symptoms, nature history, imaging tools, and optimal management of TIPIC. The current literature review is a relatively comprehensive collection of TIPIC patients available to the best of our knowledge.

The pathogenesis of TIPIC syndrome remains poorly understood. Amaravadi RR et al. performed PET-CT on patients demonstrating soft tissue enhancement at the carotid bifurcation corresponding to surrounding foci of glucose hypermetabolism, while the absence of a cervical mass and the self-limiting clinical course better explains the nature of their inflammation (7). Upton et al. performed an external biopsy of a patient with carotid pain suggesting negative Gram stain and culture, which confirmed that the thickening of the carotid artery wall is a sterile chronic inflammation (8). Gong, T et al. mention that damage-associated molecular patterns play an important role in the pathogenesis of a variety of inflammatory diseases after binding to specific receptors (9), and perhaps this pattern binds to specific receptors in the carotid artery wall causing this limited inflammation. However, conclusions drawn from a single biopsy are insufficient to establish definitive pathogenic mechanisms, and many studies are needed to confirm this, but there is no doubt that the immune system is involved.

The etiology of TIPIC syndrome remains controversial. In contrast to the statistics of cases presented in this article, most patients are in good health before the onset of the disease, with only a few having a history of respiratory infection, autoimmune disease or migraine (10) and high risk factors for hypertension and other vascular disease. Only a small proportion of reported patients demonstrated elevated serum IgM levels (2.7%). In our case, the IgM level was slightly elevated, suggesting that the TIPIC in this case might be related to nonspecific inflammation or a specific viral infection. Of interest is a case reported in the literature of carotid artery pain caused by exposure to high altitude environments (11). In another article, carotid pain is considered to be the result of a delayed hypersensitivity reaction to the breast cancer chemotherapy drug docetaxel (12). In addition, one patient developed a coexistence of TIPIC syndrome and large vessel vasculitis after granulocyte colony-stimulating factor(G-CSF) injection (13). Intramuscular of COVID-19 Vaccine also induced its onset and recurred with secondary injections (14). Additionally, biopsies are not considered to be routinely performed and due to the self-limiting nature of TIPIC syndrome, the biopsy in question may be a potential contributor to neck pain (15). This implies that the etiology of the disease is more complex than just the possibility of viral infections. The extensive previous literature is rather vague in its description of the etiology and there is no summary of it. Our review suggests that the onset of TIPIC syndrome may be multifactorial, with pharmacological, physical, infections and so on conferring this symptom. It is more likely to be a transient autoimmune abnormality caused by multiple factors. We therefore believe that all factors that can cause autoimmune disorders may lead to TIPIC syndrome under certain conditions.

Our study summarized the characteristics of TIPIC. There are few previous literatures that systematically summarizes patient symptoms. We found that pain of varying degrees, mostly located at the carotid bifurcation (72.8%), is the predominant clinical manifestation of TIPIC syndrome, often accompanied by inflammatory manifestations in the form of pressure (70.7%) and swelling (15.8%), and activities such as swallowing and coughing may exacerbate it. Some patients may experience a lymph node enlargement (9.4%) and radiating pain (10.1%) at other sites due to the involvement of the peripheral carotid plexus. Our patient had radiation to the head and pharynx, but this radiation seemed to be limited to area above the neck, and no other areas have been reported. Some patients may also experience nausea and vomiting due to severe pain, or a range of symptoms such as anxiety and irritability. Chen XW et al. found a case of hoarseness and swallowing and coughing due to inflammatory damage to the vagus and glossopharyngeal nerves in the carotid sheath (16). This suggests that when some non-characteristic manifestations other than neck pain are present, one should also be alert for concomitant symptoms of TIPIC syndrome. The ratio of male to female prevalence was previously reported in the literature as 1:1.5, our larger sample size yielded a ratio of about 1:1.4, but still prevalent in females. The median age was 48 years, which is consistent with previous reports (1).

Of the four diagnostic criteria proposed by Lecler et al, imaging should be the gold standard. The imaging of TIPIC syndrome is specific, showing amorphous soft tissue replacing the fatty formation around the carotid artery in a biased cardiovascular infiltrate (1). We found that the amorphous soft tissue most often involved the carotid bifurcation, located in the posterior lateral wall, which is consistent with previous reports (1). On CT and MRI images only the abnormal tissue wrapped around the carotid artery with enhanced signal can be seen (1), which is more obscure for the interior of the vessel. High density signal with contrast enhancement around the periphery of the carotid artery is usually seen on T1 and T2-weighted images of MRI, and the enhanced effect of T1-weighted images on peripheral fat saturation is more easily identified (17). In our case, the discrepancy between ultrasound and CTA findings was most likely attributable to the timing of imaging rather than intrinsic modality limitations. Carotid ultrasound was performed during the acute symptomatic phase and demonstrated characteristic perivascular inflammatory infiltration. In contrast, CTA was obtained after clinical improvement, at which time the inflammatory changes had already begun to resolve. This observation further supports the dynamic and self-limiting nature of TIPIC syndrome, in which imaging abnormalities may regress in parallel with symptom improvement. In our literature consolidation, 23.8% of patients presented with soft intimal plaques, and these changes in and around the arteries may be triggered by inflammation of the cholesterol-rich plaques (18), to confirm this speculation, an accurate assessment by ultrasound is indispensable. Ultrasound allows clearer localization of the vessel at the lesion site and observation of specific changes inside and outside the vessel. The lesion does not cause an increase in the circumference of the carotid artery, being eccentric because it often reaches just under half of its circumference, the lumen is mostly mildly stenosed and there is no disease or change in hemodynamics. The few reported cases of carotid pain are due to thrombosis (19), vascular variation (20) or lymph node compression (21), however these cases are not considered to be included in the diagnosis of TIPIC as there is no obvious infiltration of the surrounding vessels by adjacent adipose tissue on the imaging images. In either imaging modality, adipose tissue can be seen replacing the mesentery or epithelium, while the endothelium is mostly uninjured (17). We rule out the diagnosis of aortitis because although there is a restrictive thickening of the arterial wall, there is no significant narrowing of the vessel or increased flow rate. This thickening even disappears after a month with regular medication, a manifestation that does not occur in aortitis. Compared to CT and MRI, Ultrasound represents a practical, noninvasive, and sensitive modality for both initial evaluation and follow-up, saving patients time and expense. Moreover, as a means of follow-up, it makes it easier to observe changes in perivascular infiltration to assess the patient’s prognosis (1).

TIPIC syndrome needs to be differentiated from many other diseases. Vascular factors include Takayasu arteritis, carotid dissection and anatomic cause, etc. Non-vascular causes factors such as Eagle syndrome, lymphadenitis and thyroiditis, etc (17). These conditions may present with acute neck pain and can often be differentiated through careful history-taking and imaging evaluation, physical examination, etc. Takayasu arteritis and TIPIC syndrome have some extent similar intravascular changes, but Takayasu arteritis involves a wider range of arteries that can accumulate in the thoracic or abdominal aorta. In Takayasu arteritis, concentric, circle-like intimal thickening within affected vessels frequently leads to severe stenosis and hemodynamic compromise; these structural vascular changes are typically irreversible. In contrast, TIPIC syndrome is characterized by benign, self-limited perivascular inflammatory infiltration without documented cases of significant luminal stenosis. The hemodynamics generally do not change and the perivascular infiltration can disappear. Takayasu arteritis often shows systemic symptoms, while TIPIC symptoms are more limited. Takayasu arteritis will show a series of physical manifestations such as poor blood pressure in bilateral limbs, while TIPIC examination often only shows pressure pain or swelling in the neck (22). A paper reported that the tortuous submucosal internal carotid artery caused displacement of the tonsils resulting in neck pain, and that anatomical variants of the vessels could be clearly observed on imaging to exclude the possibility of TIPIC syndrome (20). Carotid artery entrapment can be observed on color Doppler ultrasound with the presence of a true lumen as well as a false lumen, and two-thirds of patients have a characteristic unilateral frontotemporal headache, which is relatively rare in TIPIC syndrome (23). The Eagle syndrome is a result of compression of arteries and nerves by an abnormal stromal process, the anatomical relationship with the surrounding nerves and blood vessels can be observed on CT to differentiate (24). Perivascular infiltration is not usually seen in the blood vessels of thyroiditis and lymphadenitis, etc, but are more likely to be imaging abnormalities of the lymph nodes and thyroid. In summary, the most distinctive difference is that TIPIC syndrome can present with perivascular infiltration with hardly hemodynamic disturbance, and perivascular infiltration can also disappear with pharmacological intervention or on its own. Therefore, imaging and follow-up are essential for differentiation.

TIPIC syndrome appears to be a self-limiting process and there are no guidelines for the use of medication. Non-steroidal anti-inflammatory drugs (62.2%) and steroids (18.1%) are the most common drugs used for treatment and may accelerate the disappearance of symptoms. The use of antibiotics has not been effective (25) and our patient did not experience any relief of symptoms on antibiotics, which implies that the thickening of the carotid wall is aseptic inflammation. Some patients have been given sedative-hypnotic drugs and have recovered as usual (26), one patient’s pain has not relapsed even with migraine prophylaxis (10). In Takayasu arteritis, concentric, circle-like intimal thickening within affected vessels frequently leads to severe stenosis and hemodynamic compromise; these structural vascular changes are typically irreversible. In contrast, TIPIC syndrome is characterized by benign, self-limited perivascular inflammatory infiltration without documented cases of significant luminal stenosis. The improvement in symptoms generally precedes the disappearance of the imaging manifestations and we need a large number of cases to conduct controlled studies to clarify whether medication plays a role in the disappearance of neck pain symptoms and the disappearance of perivascular infiltration. In a small proportion of patients, recurrence was observed (10.8%), sometimes involving the contralateral carotid artery. Among reported recurrent cases, several were temporally associated with vaccination or infectious exposure; however, a direct causal relationship remains speculative. The management of recurrent episodes does not appear to differ substantially from that of initial presentation (26).

## Conclusion

TIPIC is a self-limiting disease that results in chronic inflammation of localized tissues due to autoimmune abnormalities which manifests as acute neck pain and occurs mainly at the carotid artery bifurcation. The characteristic imaging features are eccentric inflammatory infiltrates in the vessel wall and surrounding tissues. Both symptoms and imaging features may resolve spontaneously or with pharmacological intervention. There are still many unanswered questions about TIPIC syndrome, such as its etiology, pathogenesis, and epidemiological features, more clinical studies will be needed to complete it in the future.

## Data Availability

The raw data supporting the conclusions of this article will be made available by the authors, without undue reservation.
